# Preparation and Evaluation of Combined Detection of Norovirus GI and GII: An Innovative Fluorescent Particles Test Strip

**DOI:** 10.1155/2018/7862467

**Published:** 2018-02-25

**Authors:** Lifang Zhang, Yanxia Peng, Na Jiang, Lei Shi, Jieping Lin, Ping Wu, Qingjun Pan

**Affiliations:** Clinical Research Center and Institute of Nephrology, Affiliated Hospital of Guangdong Medical University, Zhanjiang 524001, China

## Abstract

This study was designed to prepare and evaluate the sensitivity and specificity of a Norovirus GI and GII fluorescent particles combined detection test strip method. Using selected chromatographic materials and antibodies specific to Norovirus GI and GII, the Norovirus GI and GII fluorescent particles combined detection test strip (tested method) was prepared as a conventional double antibody sandwich. The samples assayed included cultured rotavirus and 465 specimens from patients with symptoms of gastrointestinal infection. Norovirus was detected using the tested method and a reference method (CerTest Norovirus GI-GII test card). The results indicated that the sensitivity of the tested method was 4 (for GI detection) or 8 times (for GII detection) greater than the reference method. Neither of the two methods cross-reacted with rotavirus and so on. For specimens, 29 were found to be negative by the reference method and positive by the tested method, and 8 were found to be negative by the tested method and positive by the reference method. Furthermore, a retesting of these samples by qPCR showed that 28 of the 29 were positive, and 3 of the 8 were positive. In summary, the Norovirus GI and GII fluorescent particles combined detection test strip was successfully prepared and had good detection performance.

## 1. Introduction

The nonenveloped Norovirus (NV; Caliciviridae) is composed of a single strand of RNA (27–40 nanometers diameter) [1]. NV can be divided into seven genotypes (GI, GII, GIII, GIV, GV, GVI, and GVII) based on complete amino acid sequence analysis for the capsid protein; each genotype can be divided into several subtypes [2, 3]. GI, GII, and GIV are the NV genotypes that typically infect humans. Infection with these genotypes can result in acute gastroenteritis with clinical symptoms and signs that include nausea, vomiting, watery diarrhea, stomach cramps, headache, and fever [4–6]. In developing countries, more than 200,000 deaths per year are due to NV infection. Most of the mortality occurs in children < 5 years of age, the elderly, and individuals with poor immunity [7, 8]. To prevent and control NV infection, it is urgent that a rapid, sensitive, and accurate method to detect NV should be developed and implemented.

NV can be detected using microscopy, molecular, or immunological methods [9]. The microscopy methods include direct electron and immunoelectron microscopy. These methods can only be performed by professionals, and each milliliter of sample should contain ≥1 × 10^6^ viral particles. Molecular detection of NV requires reagents and specialized instruments. Detection using molecular methods has high sensitivity and specificity [2, 8] and is usually the standard method used. Because microscopy and molecular methods require specialized equipment and professionally trained technicians, they are mainly limited to use by large hospitals. The immunological detection methods include radioimmunoassay, enzyme linked immunosorbent assay, biotin-avidin immunoassay, and test paper technology based on lateral immunochromatography. These methods can directly detect NV and do not require specialized professional test instruments or sites for use. They are used extensively in multiple fields (e.g., medicine, agriculture, animal husbandry, entry, and exit testing) [10]. The methodologies between molecular methods and immunological methods are different, such as the detective targets, as molecular methods are based on detecting specific region of RNA sequence of NV, while immunological methods are based on detecting specific antigen of NV, so the sensitivities maybe very different. Also, the sensitivity and specificity of different immunological detection methods depend on different type of tracers and the quality of paired antibodies. Detection techniques using a tracer have typically used colloidal gold for that purpose. In recent years, immunochromatography using colored latex beads has attracted significant interest from assay developers. This technique has intuitive results and can be conveniently applied, but it has not been fully developed or promoted; the market share is far less than that of colloidal gold. Very few published studies have compared the use of latex beads with other methods, including colloidal gold.

The fluorescent nanometer particle represents a new type of tracer. By combining chromatography and fluorescence detection, and by pre-preparing the detection strip, the product is easily transportable and samples can be tested at any time. After irradiation using a laser light source such as an ultraviolet lamp, a visible fluorescent band can be observed. The product has a simple design, is easy to use, and has high sensitivity [11]. Fluorescent nanometer particle detection still has some technical problems that remain to be solved, such as selection of proper fluorescent nanometer particle, antibody labeling technology, and the stable storage of labeled fluorescent nanometer particle. Relevant products are under development, but commercially available products are extremely rare. Products that offer combined detection of two targets are also uncommon. However, use of the fluorescent nanometer particle technology has very good commercial prospects.

The objectives of this study were to use fluorescent microsphere detection strips prepared in our laboratory for combined detection of NV GI and GII and to evaluate the sensitivity and specificity of the method. This product may represent a new and reliable rapid method for detection of NV GI and GII in clinical practice.

## 2. Materials and Methods

### 2.1. Materials

Fluorescent polystyrene microspheres (carboxylate-modified, 100 nm particle size, 505 nm maximal excitation wave length, 515 nm maximal emission wavelength) were obtained from Life Technologies (Rockville, MD, USA). Mouse anti-GI and anti-GII monoclonal antibodies and anti-mouse IgG polyclonal antibody were obtained from MyBioSource, Inc. (San Diego, CA, USA). Polyester cellulose membrane, nitrocellulose membrane, and PVC-bottomed plate were obtained from Shanghai Jiening Biotech Co., Ltd. (Shanghai, China). CerTest Norovirus GI-GII one step combo card test was purchased from CerTest (CerTest, Biotec, Spain). TaKaRa qPCR Norovirus (GI/GII) Typing Kit was purchased from Dalian TaKaRa Clontech (Dalian, Liaoning, China).

### 2.2. Preparation of Fluorescent Microsphere Combined Test Strip for Detection of NV GI and GII

Using the selected chromatographic materials and specific antibodies to NV GI and GII, the fluorescent microsphere combined detection strips for NV GI and GII were prepared for use as a conventional double antibody sandwich method.

**Figure 1:**
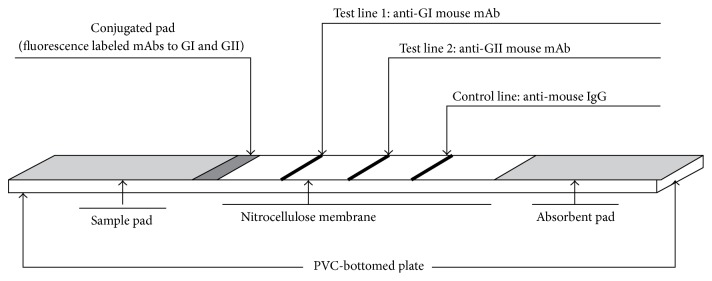
*Schematic representation of the immunochromatographic strip*. If the control line is visible, then the appearance of one or two lines of test lines indicates positive result, whereas the appearance of only the control line indicates a valid negative result.

The specific procedure was as follows:

(1) Nine-milligram fluorescent polystyrene microspheres were weighed and centrifuged at 10,000*g* for 10 min, and the sediment was collected.

(2) A 0.2 M pH9.6 bicarbonate buffer solution that was 10 times the microsphere volume was used to wash the sediment. The solution was centrifuged at 10,000*g* for 15 min, and the sediment was dispersed in 900 *μ*l 0.2 M pH9.6 bicarbonate buffer for a final microsphere concentration of 10 mg/ml.

(3) A total of 75 *μ*g each of mouse anti-GI and anti-GII monoclonal antibodies diluted with 0.01 M pH7.4 phosphate buffered saline (PBS) were added consecutively to the fluorescent microspheres dropwise. The solution was incubated at room temperature for 30 min.

(4) Bovine serum albumin (1%) was used for the block the sites on the fluorescent microspheres that did not contain bound antibody. The solution was then incubated overnight at room temperature.

(5) Working at 4°C, the fluorescent microspheres prepared in step (4) were centrifuged at 10,000*g* for 40 min to collect the sediment, washed with 0.05 M pH9.0 Tris-HCl buffer, and centrifuged. The sediment was reconstituted to the initial volume using 0.05 M pH9.0 Tris-HCl buffer to produce the fluorescent microspheres labeled with mouse anti-GI and anti-GII monoclonal antibodies.

(6) A polyester cellulose membrane was cut into 0.7 cm × 30 cm pieces. The pieces were immersed (4 *μ*l/cm^2^) in the solution prepared in step (5), then dried at 37°C for 60 min, and placed in a dry environment.

(7) A 0.01 M pH7.4 PBS solution was used to dilute mouse anti-GI and anti-GII monoclonal antibodies to 3 mg/ml. The solution was sprayed in a linear pattern onto different sites on a nitrocellulose membrane for combined detection of NV GI and GII.

(8) 0.01 M pH7.4 PBS was then used to adjust the concentration of anti-mouse IgG polyclonal antibody to 2 mg/ml. This solution was sprayed onto the chromatography membrane to serve as the quality control. The dose sprayed onto the membrane was 1.5 *μ*l/cm for all four lines; the lines were spaced at 6-mm intervals. The quality control line was 1 cm from the end of the chromatography membrane. The product was then dried overnight at 37°C and preserved in a dry environment at room temperature.

(9) A polyester cellulose membrane was immersed in 0.05 M pH9.0 Tris-HCl buffer, dried overnight at 37°C, and preserved in a dry environment at room temperature.

(10) A sample absorption mat, fluorescent microsphere binding mat, chromatographic membrane, and bibulous paper were layered onto a PVC-bottomed plate. The layers were then cut into 3-mm wide pieces to form the lateral chromatographic detection strips containing the fluorescent microspheres.

(11) Schematic for the final product (Figure 1).

(12) Finally, the lateral chromatographic detection strips were fixed in a plastic shell (Figure 2).

### 2.3. Samples

Specimens and cultures of common intestinal viruses that infect humans are preserved in our laboratory. The NV (Wa strain) is included in this collection. Clinical stool specimens were collected from 465 patients with symptomatic gastrointestinal infection. The patients were examined at our hospital and the Guangzhou Women and Children Medical Care Center. The sample population included 309 males and 156 females (mean age, 12.7 ± 9.3 years).

### 2.4. Methods

#### 2.4.1. Principle

During use of the fluorescent microsphere test strip for combined detection of NV GI and GII, the results were assessed as per the manufacturer's instructions. The manufacturers' instructions were also followed for the CerTest Norovirus GI-GII one step combo card test and the TaKaRa qPCR Norovirus (GI/GII) Typing Kit.

#### 2.4.2. Sensitivity Evaluation

Specimens of strong positive NV GI (three specimens) or GII (three specimens) results or negative results (three specimens) detected with three methods were collected, mixed thoroughly as one sample, respectively, and then used for sensitivity analysis. PBS was used for double dilution, and then the combined detection fluorescent microsphere test strip (tested method) and the CerTest Norovirus GI-GII test (reference method) were each used to detect NV.

#### 2.4.3. Evaluation of Specificity

The two methods including fluorescent microsphere test strip and CerTest Norovirus GI-GII test were used to detect cell cultures of rotavirus (Wa strain), Enteric adenovirus (type 40), Coxsackievirus (A16), Echovirus (type 30), and Enterovirus (EV71).

#### 2.4.4. Detection of Clinical Specimens

Sterile bamboo sticks were used to select fresh stool specimens with mucus, pus, and blood. These samples were prepared as test samples after they were stirred and were dispersed in the dilution solution: for liquid stool specimens or liquid-like stool specimens, without dilution; for solid sample, diluted with 0.01 M pH7.4 PBS to 5–10% (g/V). Then, about 100 *μ*l (about 2-3 drops) of sample was taken into the test strip with a pipette, and the test results were read in 5–15 min.

### 2.5. Statistical Analysis

Multiple group comparison was performed using one-way ANOVA, followed by Bonferroni or Dunnett post hoc tests, and, if significance was reached, an unpaired two-tailed Student's *t*-test was performed between each compared population, unless otherwise indicated. *P* < 0.05 was considered statistically significant. Statistical analysis was performed with SPSS 15.0.

## 3. Results

The fluorescent microsphere combined test strip for NV GI and GII was successfully prepared. The schematic for the lateral chromatographic detection strips was shown in Figure 1, and the lateral chromatographic detection strips fixed in a plastic shell and the interpretation of test results were shown in Figure 2.

### 3.1. Detection Efficiency

#### 3.1.1. Sensitivity

When the GI/GII NV mixed positive specimens were diluted to 1/2^7^ and 1/2^6^, the CerTest Norovirus GI-GII one step combo card test (reference method) results were negative. The fluorescent particles test strip (tested method) results were positive for detection of NV. The sensitivity of the tested method was 4 (for GI detection) or 8 times (for GII detection) greater than the reference method (Table 1). For the results of different dilutions of negative samples, both methods showed negative results.

#### 3.1.2. Specificity

The test results indicated that the reference and tested method did not cross-react with rotavirus (Wa strain), Enteric adenovirus (type 40), Coxsackievirus (A16), Echovirus (type 30), or Enterovirus (EV71); both had good specificity towards NV GI and GII.

### 3.2. Detection of Clinical Specimens

The fluorescent particles test strip (tested method) and the CerTest Norovirus GI-GII one step combo card test (reference method) were used to detect virus in the same 465 stool specimens. The results showed that 156 (33.5%) were GI-GII positive and 309 (66.5%) were negative by tested method, and 135 (29.0%) were GI-GII positive and 330 (71.0%) were negative by reference method. In addition, 127 (27.3%) specimens were positive and 301 (64.7%) were negative as detected by both tested method and reference method (Table 2). The different test results of stool specimens by tested method and reference method were retested using the TaKaRa qPCR Norovirus (GI/GII) Typing Kit (Table 3). The results showed that 18 specimens had nucleic acids positive for GI virus and 10 had nucleic acids positive for GII virus, with only 1 negative in 29 specimens, which was negative result using the reference method but positive antigen result (Table 2). Also, 5 specimens had nucleic acid negative for GI/GII virus and 3 had nucleic acid positive for GII virus in 8 specimens, which was negative result using the tested method and positive result using the reference assay (Table 2).

## 4. Discussion

Worldwide, NV outbreaks occur in various locations each year. Populations in countries that are less economically advanced are at greatest risk of clinical NV infection. The management of NV diarrhea, which is similar to rotaviruses, falls within the multifaceted programmed for the management of diarrhea disease which include rehydration. Individuals infected with NV cannot be rapidly diagnosed because a rapid, sensitive, accurate, and economic detection method is not available. The lack of a rapid diagnosis results in deterioration of the patient's condition; mortality can occur in severely affected patients [7, 8]. Although virus vaccines are available to prevent and control NV infection, these vaccines have limited efficacy [8, 12] and limited duration of protection (i.e., 6–9 months) [8, 13]. Products to detect virus using colloidal gold as the tracer have been extensively used for detection of NV infection [14], but products for combined detection of GI/GII virus are extremely rare. The CerTest Norovirus GI-GII one step combo card test is more frequently used by departments of laboratory investigation, but colored microsphere and colloidal gold techniques have low sensitivities. There is a high risk that a false negative result will be obtained, particularly when the stool specimen contains a low concentration of virus.

In this study, we used the selected chromatographic materials and specific antibodies to prepare a fluorescent microsphere combined detection strip for NV GI and GII. Specifically, the antibodies attached on the microspheres were through formation of a peptide bond (-CO-NH- bond) by loss of water from two amino acids. Using the CerTest Norovirus GI-GII one step combo card test produced by CerTest in Spain as the reference test, we evaluated the performance of the prepared fluorescent microsphere combined detection strip. The results showed that out of the total number of samples tested, 29 were found to be negative by the CerTest Kit and 8 negative by the tested method. According to the retested results using the TaKaRa qPCR Norovirus (GI/GII) Typing Kit, 28 of the 29 CerTest Kit negatives were false negatives whilst only 5 of the 8 the microsphere combined tested samples were false negatives. Taken together, these results indicated that the prepared fluorescent microsphere combined detection strip had a significantly higher sensitivity than that of the colored microsphere method. The fluorescent microsphere combined detection strip did not cross-react with other common intestinal viruses. The prepared fluorescent microsphere detection strip rapidly and simultaneously detected NV GI and GII and can be used to provide timely and reliable results for clinical diagnosis.

In summary, in this study we prepared fluorescent microsphere combined detection strips for NV GI and GII. This assay had good detection performance, is simple and rapid to use, and could be used by clinical facilities and in the field for detection of NV. This product is a more convenient and sensitive method for detection of NV GI and GII.

## Figures and Tables

**Figure 2:**
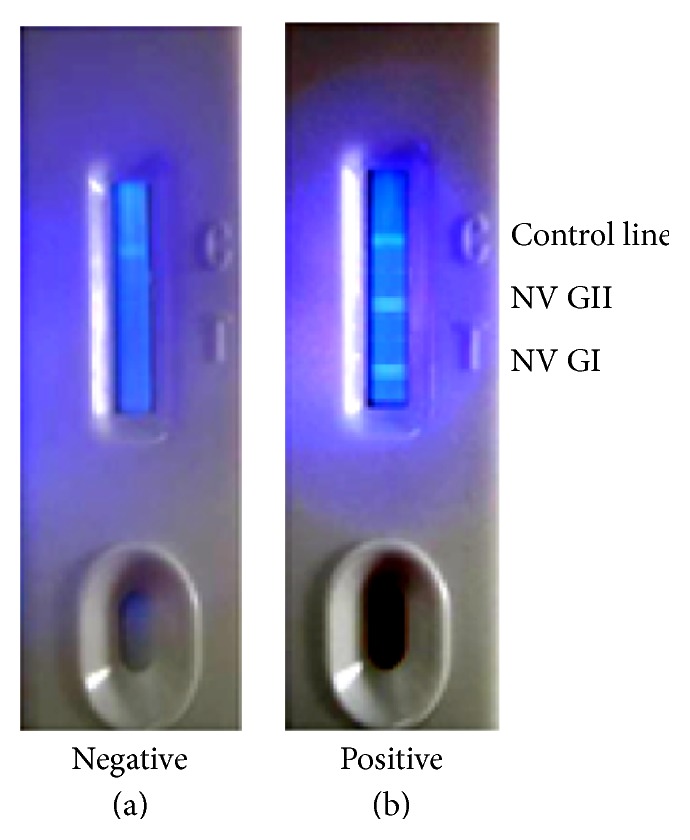
*The fluorescent microsphere combined test strip for NV GI and GII*. Interpretation of test results: control line, and test line (NV GI and GII); after excitement by the laser, (a) indicates negative results, and (b) indicates double-positive results (a sample tested was a dual infection).

**Table 1:** Comparison of the test results of the two methods using different dilutions of Norovirus GI/GII positive stool specimens.

	Serial dilutions								
	1/2	1/2^2^	1/2^3^	1/2^4^	1/2^5^	1/2^6^	1/2^7^	1/2^8^	1/2^9^
Norovirus GI									
Reference method	+	+	+	+	+	+	−	−	−
Tested method	+	+	+	+	+	+	+	+	−
Norovirus GII									
Reference method	+	+	+	+	+	−	−	−	−
Tested method	+	+	+	+	+	+	+	+	−

**Table 2:** Comparison of the tested results for the two methods used to detect Norovirus in stool specimens.

	Reference method		Total (*n*, %)
	Positive	Negative	
Tested method			
Positive	127	29	156 (33.5%)
Negative	8	301	309 (66.5%)

Total (*n*, %)	135 (29.0%)	330 (71.0%)	465 (100%)

**Table 3:** Different test results for 37 stool specimens by the two methods were retested using the TaKaRa qPCR Norovirus (GI/GII) Typing Kit.

	qPCR Norovirus (GI/GII) Typing Kit			Total
	GI positive	GII positive	Negative	
Tested method				
Positive	18	10	1	29
Negative	0	3	5	8

Total	18	13	6	37
